# Clinical Outcomes After Hysteroscopic Removal of Retained Products of Conception with or Without Prior Uterine Artery Embolization

**DOI:** 10.3390/jcm14228020

**Published:** 2025-11-12

**Authors:** Eva Skuk, Polona Vihtelič, Peter Popovič, Kaja Kovač, Ivan Verdenik, Nataša Kenda Šuster

**Affiliations:** 1 Department of Gynaecology, University Medical Centre Ljubljana, 1000 Ljubljana, Slovenia; eva.skuk@gmail.com (E.S.);; 2 Faculty of Medicine, University of Ljubljana, 1000 Ljubljana, Slovenia; 3 Clinical Institute of Radiology, University Medical Centre Ljubljana, 1000 Ljubljana, Slovenia

**Keywords:** retained products of conception, uterine artery embolization, hysteroscopy, fertility, high vascularity, reproductive outcomes

## Abstract

**Purpose**: Retained products of conception (RPOC) are a common complication after pregnancy. While hysteroscopic resection is the standard treatment when RPOC does not resolve spontaneously, highly vascular tissue can lead to severe bleeding during the procedure. This study assessed clinical outcomes, procedural safety, and reproductive performance in patients with highly vascular RPOC treated with uterine artery embolization (UAE) prior to hysteroscopy, compared to those treated with hysteroscopy alone. **Methods**: This retrospective study included 42 women diagnosed with RPOC at University Medical Centre Ljubljana, Slovenia (2010–2020). Patients were divided into two groups: UAE followed by hysteroscopic resection (UAE + HSC, *n* = 21) and hysteroscopic resection alone (HSC-only, *n* = 21). Data on clinical outcomes, complications, and reproductive history were analyzed using Fisher’s exact and *t*-tests (*p* < 0.05). **Results**: Groups were similar in baseline characteristics, except for greater vascularity in the UAE + HSC group (100% vs. 4.8%, *p* < 0.05). Bleeding >300 mL occurred more often in the UAE + HSC group, but all cases were managed conservatively and only one patient required transfusion. No uterine perforations occurred. Residual RPOC was found in one patient per group. Rates of endometritis, menstrual changes, and pelvic pain were comparable. Among those who attempted conception, live birth and miscarriage rates did not differ significantly. **Conclusions**: UAE prior to hysteroscopic surgery appears to be a safe and effective option for highly vascular RPOC, especially in patients at risk of hemorrhage, with no adverse impact on fertility. Further prospective studies are recommended.

## 1. Introduction

Retained products of conception (RPOC) refer to the persistence of trophoblastic tissue within the uterine cavity following pregnancy termination, miscarriage, or delivery—either vaginal or cesarean [1,2]. The presence of RPOC can lead to a range of clinical complications. In the acute setting, patients may experience abnormal uterine bleeding, hemorrhage, pelvic pain, or infection. Long-term complications include intrauterine adhesions, menstrual irregularities, new onset pelvic pain, infertility, recurrent pregnancy loss, and abnormal placentation in subsequent pregnancies [1]. The prevalence of RPOC is estimated to be approximately 1% following term pregnancies, with a higher incidence observed after medical termination of pregnancy or spontaneous miscarriage [3]. Recent studies have described a distinct subtype of RPOC characterized by pronounced vascularity detected by Doppler ultrasonography. The reported incidence of this highly vascular variant is approximately 18% among all RPOC [4].

Transvaginal ultrasound, particularly when supplemented with color Doppler imaging, is the diagnostic modality of choice due to its high sensitivity in evaluating tissue vascularity [3]. While dilation and curettage has traditionally been the standard therapeutic approach, associated risks—including uterine perforation, incomplete evacuation, and intrauterine adhesions—have prompted a shift towards hysteroscopic techniques. Hysteroscopy (HSC) allows direct visualization of the uterine cavity and facilitates more complete and targeted removal of retained tissue [2].

In cases where RPOC exhibits significant vascularity (Doppler color scores 3 and 4), there is a high risk of intraoperative hemorrhage, which in rare cases can necessitate hysterectomy. In such instances, preoperative uterine artery embolization (UAE), a minimally invasive technique, can be performed to reduce the vascular supply to the retained tissue, thereby lowering the risk of severe hemorrhage during surgery and preserving future fertility [3]. UAE involves selective occlusion of the uterine arteries using various embolic agents, including microspheres, gelatin sponge, liquid embolics, and cyanoacrylate adhesives. In our clinical practice, gelatin sponge is the preferred embolic material due to its temporary occlusive effect—lasting approximately 3 to 6 weeks—which allows for subsequent recanalization and is considered advantageous in patients desiring future fertility [3].

Although UAE is an established intervention for conditions such as postpartum hemorrhage and uterine fibroids, its role in the management of RPOC remains an area of ongoing investigation [1]. The objective of this study is to present our clinical experience by assessing procedural outcomes, complications, and subsequent reproductive and obstetric results in patients undergoing UAE followed by selective hysteroscopic resection for the treatment of highly vascular RPOC.

## 2. Methods

This retrospective cohort study included 42 women diagnosed with RPOC who were treated with hysteroscopy at the Department of Gynecology, University Medical Centre Ljubljana, between January 2010 and December 2020. Eligible patients presented with persistent or delayed vaginal bleeding following early pregnancy loss, including spontaneous abortion or termination of pregnancy initially managed with a mifepristone–misoprostol regimen.

### 2.1. Diagnosis

**Figure 1:**
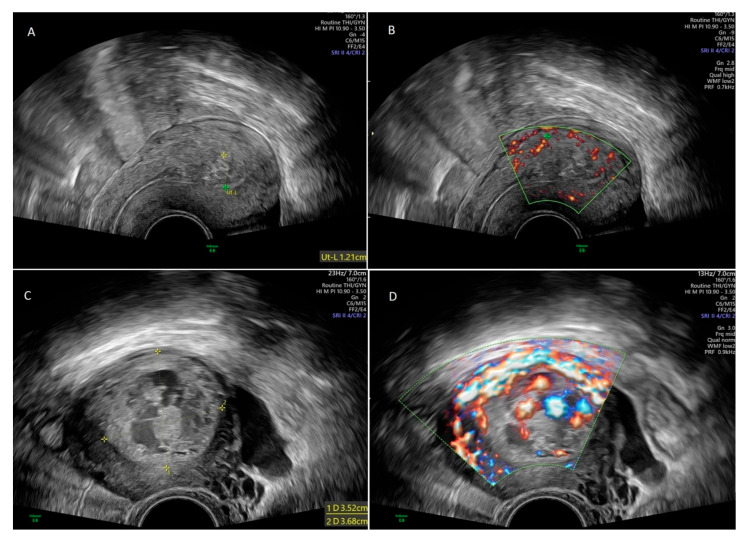
Transvaginal ultrasonography in 30-year old female with retained products of conception (RPOC) (**A**). Color Doppler ultrasound in the same patient showed minimal vascularity of the lesion (**B**). Transvaginal ultrasonography in 31-year old female with RPOC (**C**). Color Doppler ultrasound in the same patient showed hypervascularity of the lesion (**D**).

The diagnosis of RPOC was established based on clinical symptoms such as abnormal bleeding and transvaginal ultrasonography supplemented with color Doppler imaging. Sonographic criteria for RPOC included the presence of a heterogeneous intracavitary, predominantly hyperechoic focal mass, with or without increased vascularity [2]. Hypervascularity was classified based on color Doppler indices (color score 3 or 4). Transvaginal ultrasound in two RPOC cases are shown in Figure 1: one with minimal vascularity of the lesion and one case with highly vascular RPOC.

### 2.2. Study Groups

The study included women diagnosed with retained products of conception (RPOC) confirmed by ultrasound and color Doppler after early pregnancy loss or termination of pregnancy, treated by hysteroscopic resection. Exclusion criteria included RPOC treated by dilatation and curettage, which is a blind technique.

Patients were categorized into two groups based on treatment modality. The study group (UAE + HSC, *n* = 21) included all patients who underwent uterine artery embolization prior to hysteroscopic resection. Referral for UAE was made for cases with highly vascular RPOC (color Doppler score 3–4), as confirmed by a multidisciplinary clinical team. The control group (HSC, *n* = 21) comprised patients who underwent hysteroscopic resection only, without prior embolization; in this group, the degree of vascularization was low (color score 0–2). One patient with highly vascular RPOC who declined embolization was included in the hysteroscopy-only group. This grouping reflects clinical decision-making rather than random allocation, as embolization of low-vascular cases would not have been ethically justified.

### 2.3. Uterine Artery Embolization Procedure

**Figure 2:**
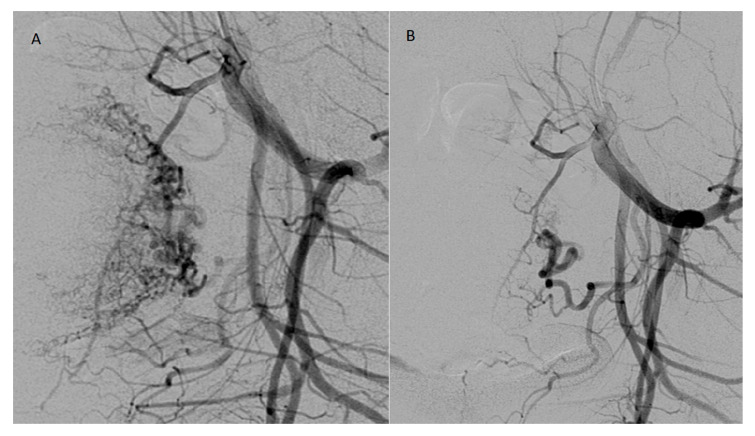
Right uterine arteriogram in the patient before prophylactic embolisation with absorbable gelatin sponge particles (**A**). Postembolisation uterine arteriogram demonstrating successful embolization (**B**).

Uterine artery embolization (UAE) was performed by experienced interventional radiologists using standard angiographic techniques under local anesthesia. Importantly, embolization was performed in a targeted and selective manner, occluding only the arterial branches supplying the retained tissue rather than the entire uterine arteries. This selective approach, achieved with a microcatheter, minimized the risk of ischemic complications. Absorbable gelatin sponge particles (Marbagelan) were used as the main embolic agent, providing temporary occlusion (3–6 weeks) and allowing arterial recanalization, thereby helping preserve uterine perfusion and fertility. The procedure was technically successful in all cases, and patients were monitored for 12–24 h for possible complications such. Possible complications related to the procedure itself and during the postoperative period include bleeding from the puncture site, ischemic or cramping pain during or after the procedure, infection, allergic reaction [5,6]. No major adverse events were observed. Figure 2 shows uterine angiography before and after UAE.

### 2.4. Hysteroscopic Resection

All patients underwent HSC removal of RPOC using a rigid hysteroscope with saline distension under general anesthesia. Tissue resection was performed using either a mechanical or bipolar resection loop. Complete clearance of retained tissue was confirmed by direct visualization of the uterine cavity. In the UAE + HSC group, HSC was performed within 24 to 72 h after embolization. All patients received prophylactic antibiotics.

### 2.5. Data Collection and Outcome Measures

Baseline characteristics, including demographic and clinical data, were retrieved from hospital records and operative notes. Long-term follow-up was conducted via chart review and structured telephone interviews at least 12 months post-treatment. Variables collected included patient age, parity, previous cesarean section, first and second trimester abortions, gestational age at current pregnancy loss or termination, time from the end of pregnancy to intervention, ultrasonographically measured size of RPOC, and its Doppler vascular grading. Perioperative outcomes included complications such as estimated intraoperative blood loss, uterine perforation, postoperative infection, and the need for blood transfusion. Secondary outcomes encompassed *new-onset* pelvic pain, menstrual irregularities, fertility outcomes (spontaneous conception, conception achieved with assisted reproductive technology (ART), miscarriage), and obstetric outcomes (live births, vaginal and cesarean deliveries, gestational age at birth, preterm deliveries, birth weight, postpartum hemorrhage, manual removal of placenta) in subsequent pregnancies. The study was approved by the Republic of Slovenia National Medical Ethics Committee on 11 November 2021 (approval number: 0120-285/2021/11) and was conducted in accordance with the principles of the Declaration of Helsinki. Informed consent was obtained from all subjects involved in the study.

### 2.6. Statistical Analysis

Descriptive statistics were used to summarize patient characteristics and outcomes. Categorical variables were compared between groups using Fisher’s exact test (small sample size). Continuous variables were analyzed using independent samples *t*-tests when data were normally distributed, as assessed by mean and standard deviation. Where applicable, results are reported as mean ± SD with two-tailed *p*-values. A *p*-value < 0.05 was considered statistically significant. All analyses were performed using SPSS Statistics (version 28.0).

## 3. Results

A total of 42 patients with RPOC were included in the present study: 21 patients in the study group underwent UAE followed by HSC surgery (UAE + HSC group), and 21 patients in the control group underwent HSC surgery alone (HSC only group).

### 3.1. Baseline Characteristics

Baseline characteristics were similar between the groups. The mean age was 32.8 ± 5.7 years in the UAE + HSC group and 31.9 ± 6.1 years in the HSC group (*p* = 0.62). Parity, history of previous cesarean section, and previous first or second trimester abortions were comparable across both groups. The mean interval from pregnancy termination to treatment for RPOC did not differ significantly (34.5 ± 22.8 vs. 38.2 ± 25.1 days, *p* = 0.62). The dimensions of RPOC were 27.1 ± 10.9 mm × 22.8 ± 7.5 mm in the UAE + HSC group and 22.9 ± 7.2 mm × 19.3 ± 8.1 mm in the HSC-only group. Notably, all patients in the UAE + HSC group demonstrated high vascularity on Doppler ultrasound (Color Doppler score 3 or 4), compared to only one patient in the HSC-only group (100% vs. 4.8%, *p* < 0.05). Data are shown in Table 1.

### 3.2. Surgical Outcomes and Complications

Significant intraoperative bleeding (>300 mL) occurred in 5 patients (23.8%) in the UAE + HSC group compared to 2 patients (9.5%) in the HSC-only group (*p* = 0.41). All cases of intraoperative bleeding were managed conservatively. Postoperative blood transfusion was required in 1 patient (4.8%) in the UAE + HSC group, while no patients (0%) in the HSC-only group required transfusion. No uterine perforations occurred in either group. Residual RPOC was detected in 1 patient (4.8%) in each group. Rates of endometritis (19.0% in both groups), menstrual irregularities (42.9% in both groups), and *new-onset* pelvic pain (33.3% in UAE + HSC vs. 19.0% in HSC-only, *p* = 0.48) did not differ significantly. Data are shown in Table 2.

### 3.3. Fertility and Pregnancy Outcomes

Thirteen patients in each group planned to conceive (61.9%). Among them, conception was achieved by 12 patients (92.3%) in the UAE + HSC group and all 13 patients (100%) in the HSC-only group (*p* = 1). Most conceptions were spontaneous, with ART used in 1 case (7.7%) in the UAE + HSC group and 3 cases (23.1%) in the HSC-only group (*p* = 0.59). The mean time to conception was 11 ± 7 months in the UAE + HSC group and 9 ± 6 months in the HSC-only group (*p* = 0.45). Although miscarriage rates differed (23.1% vs. 7.7%), this difference was not statistically significant (*p* = 1). Rates of recurrence of RPOC after abortion or delivery were comparable between groups (7.7% vs. 15.4%, *p* = 1).

Live birth occurred in 76.9% of conceptions in the UAE + HSC group and 92.3% in the HSC-only group (*p* = 0.593). Cesarean delivery was more common in the UAE + HSC group than in the HSC-only group (40.0% vs. 25.0%); however, this difference was not statistically significant (*p* = 1). Gestational age at birth (266.5 ± 17 days vs. 275.8 ± 16 days) did not differ significantly between the groups (*p* = 0.17). The preterm delivery before 34 weeks was also comparable (10.0% in UAE + HSC vs. 8.3% in HSC-only group). In the UAE + HSC group, preterm birth was attributed to preterm premature rupture of membranes (PPROM), whereas in the HSC-only group, it was due to medically indicated induction for preeclampsia. Birth weight was similar between groups (3053 ± 720 g vs. 3080 ± 716 g, *p* = 0.95). Rates of postpartum hemorrhage (38.5% in both groups) and manual removal of the placenta (23.1% vs. 25.0%) were also comparable. Data are shown in Table 3.

## 4. Discussion

This study evaluated the clinical impact of UAE followed by HSC surgery in the management of highly vascular RPOC compared to HSC alone. Our findings demonstrate that UAE is a safe and effective adjunct for managing patients at high risk of hemorrhage due to increased vascularity of RPOC. Although UAE aims to reduce intraoperative bleeding, patients in the UAE + HSC group still experienced higher mean blood loss and a greater incidence of bleeding compared to the control group. This likely reflects the inherently more vascular nature of RPOC in the UAE group rather than a limitation of the embolization procedure itself. Importantly, UAE did not negatively affect reproductive or obstetric outcomes, with similar rates of conception, live birth, and obstetric complications observed in both groups. These results suggest that while UAE may not completely prevent intraoperative bleeding, it helps make bleeding manageable and remains a safe, fertility-preserving option for selected patients with highly vascular RPOC.

As previously mentioned, we observed a higher, though not statistically significant, incidence of severe intraoperative bleeding (>300 mL) during HSC surgery in the UAE + HSC group compared to the HSC-only group. This finding aligns with the study by Ohmaru-Nakanishi et al. (2019), which reported similar intraoperative bleeding rates in patients undergoing HSC with or without prior UAE [1]. The increased bleeding in the UAE + HSC group can primarily be attributed to the fact that all patients in this group had a high color Doppler score (CS 3 or 4), indicating increased vascularity of the RPOC, compared to a significantly lower incidence of high vascularity in the HSC-only group. This observation is supported by previous research showing that increased vascularity of RPOC correlates with a higher risk of hemorrhage during surgical intervention [2]. The higher bleeding rate in the UAE + HSC group also underscores concerns about altered vascularization, possibly involving arteriovenous malformations between the RPOC and uterine wall, as noted by Bazeries et al. (2017) [3]. Another contributing factor to the increased bleeding may be the larger volume of residual tissue in the UAE + HSC group. Importantly, despite the higher bleeding risk, all cases were managed successfully with conservative measures such as uterotonics, without the need for additional surgical interventions or even hysterectomy. Postoperative blood transfusion was required in only one patient in the UAE + HSC group, while none were needed in the HSC-only group. The rate of reintervention due to residual intracavitary tissue was low and identical in both groups, with one patient in each requiring further treatment.

Postoperative complications, including endometritis (19.1%) and menstrual cycle irregularities (42.9%), were observed at similar rates in both groups. All patients received a single dose of prophylactic perioperative antibiotics, with some cases receiving extended treatment based on clinical indications. The identical rates of endometritis suggest that UAE did not increase the risk of postoperative infection. Although the rate of menstrual disturbances was relatively high, it was comparable between groups. According to the literature, UAE may compromise endometrial integrity due to transient or localized ischemia affecting both the endometrium and myometrium, potentially resulting in menstrual irregularities [7,8]. Mechanical and especially thermal injury to the basal endometrium and underlying myometrium during HSC resection can itself cause menstrual disturbances. New-onset pelvic pain was reported more frequently in the UAE + HSC group (33.3%) compared to the HSC-only group (19.0%), though this difference was not statistically significant. This trend may be associated with post-embolization syndrome or subclinical ischemic effects, which have been described following embolization in other gynecologic procedures [9,10].

Encouragingly, the study demonstrated a high conception success rate among patients desiring fertility, with the majority of these conceptions occurring spontaneously (92.3% vs. 76.9%), underscoring that UAE does not appear to compromise reproductive capacity. These findings are consistent with previous reports, which similarly observed no significant reduction in fertility following UAE [9,10]. Furthermore, the mean time to conception was comparable between the UAE + HSC group (11 ± 7 months) and the HSC-only group (9 ± 6 months), providing additional evidence for the preservation of reproductive potential after embolization.

Early pregnancy loss within the first year after treatment was observed in 23.1% of cases in the UAE + HSC group and 7.7% of cases in the HSC-only group. The rate observed in the HSC-only group aligns with prior studies on reproductive outcomes after RPOC treatment, which reported a 6.9% incidence of early pregnancy loss following hysteroscopic removal of RPOC [11]. Although the rate in the UAE + HSC group was approximately three times higher, this difference was not statistically significant. The higher incidence in the UAE + HSC group may be related to transient or localized ischemia caused by UAE, which could affect the endometrium and myometrium and potentially increase the risk of early pregnancy loss. Recurrent RPOC was identified more frequently in the HSC-only group (2 cases) compared to the UAE + HSC group (1 case), though the difference was not statistically significant. Existing literature indicates that a prior history of RPOC is a significant risk factor for recurrence [4,12].

Pregnancy-related complications occurred at similar rates in both groups. Gestational age at birth was comparable (266.5 ± 17 days vs. 275.8 ± 16 days) and consistent with the average gestation in Slovenia, which is 39 weeks. There were two preterm deliveries before 34 weeks, one in each group. In the UAE + HSC group, the preterm birth was attributed to PPROM, while in the HSC-only group, it was due to medically indicated induction for preeclampsia. PPROM is commonly associated with intrauterine infection, whereas preeclampsia typically results from placental abnormalities caused by inadequate blood vessel supply. Birth weights were similar between the two groups (3053 g vs. 3080 g), although both were below the Slovenian average of 3400 g (Perinatal Information System of the Republic of Slovenia), which is most likely due to abnormal placentation as seen in case of RPOC.

Postpartum hemorrhage occurred in 38.5% of patients in both groups, and manual removal of the placenta was required in 23.1% versus 25% of cases, respectively. These rates are substantially higher than those reported in the general obstetric population, where postpartum hemorrhage occurs in 4–13% of cases and manual removal of the placenta in 0.3–2% of cases [13,14]. These elevated rates, as already mentioned, more likely reflect an underlying predisposition of abnormal placentation and delivery complications in patients with a history of RPOC, than previous UAE treatment. This interpretation aligns with findings from other studies reporting increased obstetric morbidity in similar clinical contexts [15].

This study was conducted at a tertiary center with an experienced team but is not without limitations. Our study included women with ultrasound- and color Doppler–confirmed retained products of conception (RPOC) following early pregnancy loss or termination, treated by hysteroscopic resection. Patients with highly vascular RPOC (color Doppler score 3–4) underwent uterine artery embolization prior to hysteroscopy (UAE + HSC group), while those with low or absent vascularity (score 0–2) underwent hysteroscopic resection only. One patient with highly vascular RPOC who declined embolization was included in the hysteroscopy-only group. This grouping reflected clinical decision-making rather than randomization, as embolization of low-vascular cases would not have been ethically appropriate. Although this heterogeneity represents a limitation, random allocation was not feasible due to the substantially higher hemorrhagic risk in hypervascular RPOC, where embolization is required for safe management. Consequently, the study reflects real-world clinical practice rather than experimental randomization. The aim was not to compare identical cohorts but to demonstrate that selective UAE effectively prevents severe bleeding in highly vascular RPOC without compromising reproductive or obstetric outcomes. While intraoperative bleeding was more frequent in the UAE + HSC group due to higher baseline vascularity, all cases were managed conservatively, and only one transfusion was required. No major complications occurred, supporting the role of UAE as a preventive rather than a causative factor in hemorrhage.

Furthermore, the retrospective design inherently introduces potential selection and information biases. The relatively small sample size—attributable to the rarity of the UAE procedure, which is performed only at this center in Slovenia—may limit the generalizability of the results. Additionally, the follow-up duration may have been insufficient to fully capture long-term reproductive and obstetric outcomes, including late-onset complications or delayed conception. To strengthen these conclusions and support clinical decision-making, future prospective studies involving larger, multicenter cohorts with comprehensive patient characterization and longer follow-up periods are essential.

## 5. Conclusions

Our findings suggest that UAE is a clinically effective adjunct in the management of highly vascular RPOC, particularly in patients at increased risk of hemorrhage. Importantly, UAE does not appear to negatively impact subsequent fertility or obstetric outcomes. Despite these encouraging results, UAE should be reserved for cases of markedly highly vascular RPOC where the benefits clearly outweigh the potential risks to future fertility or obstetric complications. Although limited by a small sample size, this study provides valuable insights to support clinical decision-making. Further large-scale, prospective studies are needed to better define the long-term reproductive and obstetric outcomes associated with UAE in patients with highly vascular RPOC.

## Figures and Tables

**Table 1:** Baseline characteristics of study participants.

Characteristic	UAE + HSC (n = 21)	HSC Only (n = 21)	*p*-Value
Age (years), mean ± SD	32.8 ± 5.7	31.9 ± 6.1	0.62
Parity, mean ± SD	1.0 ± 0.6	1.1 ± 0.7	0.62
Previous cesarean section (n, %)	3 (14.3%)	2 (9.5%)	1
Previous 1st and 2nd trimester abortion (n, %)	16 (76.2%)	15 (71.4%)	1
Time from termination of pregnancy to treatment of RPOC (days), mean ± SD	34.5 ± 22.8	38.2 ± 25.1	0.62
Size (mm) of RPOC on ultrasound (length × heigh) mean ± SD	27.10 ± 10.85 × 22.76 ± 7.51	22.86 ± 7.26 × 19.29 ± 8.11	0.09 *
High vascularity of RPOC (Color Doppler score 3 or 4) (n, %)	21 (100%)	1 (4.8%)	<0.05

*p*-values for categorical variables were calculated using Fisher’s exact test. Continuous variables were analyzed using independent samples *t*-test. Statistical significance was set at *p* < 0.05. * The combined *p*-value for both length and height measurements. Abbreviations: SD: standard deviation, HSC: hysteroscopic removal of tissue, UAE: uterine artery embolization; RPOC: retained products of conception.

**Table 2:** Surgical outcomes and complications.

Outcome	UAE + HSC (n = 21)	HSC Only (n = 21)	*p*-Value
Bleeding (>300 mL)	5 (23.8%)	2 (9.5%)	0.41
Postoperative transfusion	1 (4.8%)	0 (0%)	-
Uterine perforation during HSC procedure	0 (0%)	0 (0%)	1
Residual RPOC	1 (4.8%)	1 (4.8%)	1
Endometritis	4 (19.0%)	4 (19.0%)	1
Menstrual irregularities	9 (42.9%)	9 (42.9%)	1
Chronic pelvic pain	7 (33.3%)	4 (19.0%)	0.48

*p*-values for categorical variables were calculated using Fisher’s exact test. Continuous variables were analyzed using independent samples *t*-test. Statistical significance was set at *p* < 0.05. Abbreviations: HSC: hysteroscopic removal of tissue, UAE: uterine artery embolization; RPOC: retained products of conception.

**Table 3:** Fertility and pregnancy outcomes following treatment.

Pregnancy Outcome	UAE + HSC	HSC Only	*p*-Value
Patients planning conception	13/21 (61.9%)	13/21 (61.9%)	1
Spontaneous conceptions	12/13 (92.3%)	10/13 (76.9%)	1
Conceptions via ART	1/13 (7.7%)	3/13 (23.1%)	0.59
Mean time to conception (months) mean ± SD	11 ± 7	9 ± 6	0.45
Miscarriages	3/13 (23.1%)	1/13 (7.7%)	1
Recurrent RPOC after abortion/delivery	1/13 (7.7%)	2/13 (15.4%)	1
Live births	10/13 (76.9%)	12/13 (92.3%)	0.59
Vaginal deliveries	6/10 (60.0%)	9/12 (75.0%)	0.65
Cesarean deliveries	4/10 (40.0%)	3/12 (25.0%)	0.65
Gestational age at birth (days) mean ± SD	266.5 ± 17	275.8 ± 16	0.17
Preterm delivery (<34 weeks of gestation)	1/10 (10%)	1/12 (8.3%)	1
Birth weight (grams) mean ± SD	3053 ± 720	3080 ± 716	0.95
Postpartum hemorrhage (PPH)	5/10 (38.5%)	5/12 (41.7%)	1
Manual removal of placenta	3/10 (23.1%)	3/12 (25%)	1

*p*-values for categorical variables were calculated using Fisher’s exact test. Continuous variables were analyzed using independent samples *t*-test. Statistical significance was set at *p* < 0.05. Abbreviations: SD: standard deviation, HSC: hysteroscopic removal of tissue, UAE: uterine artery embolization; RPOC: retained products of conception; ART: assisted reproductive technologies; PPH: postpartum hemorrhage.

## Data Availability

Data are available from the corresponding author upon reasonable request.
